# Method for Compensating Signal Attenuation Using Stepped-Frequency Ground Penetrating Radar

**DOI:** 10.3390/s18051366

**Published:** 2018-04-27

**Authors:** Tao Liu, Yutao Zhu, Yi Su

**Affiliations:** School of Electronic Science, National University of Defense Technology, Changsha 410073, China; zhu_yu_tao@yeah.net (Y.Z.); y.su@yeah.net (Y.S.)

**Keywords:** ground penetrating radar, subsurface sensor, stepped-frequency continuous wave, attenuation compensation, pseudo time–frequency transform

## Abstract

Ground penetrating radar (GPR) is a subsurface remote sensor that allows the user to detect, classify, and identify the buried target and structures. The radar signals are rapidly attenuated as they propagate into the ground; therefore, attenuation compensation is necessary for the visualization of the buried targets from GPR data. In this work, we developed a novel attenuation compensation approach based on the recently developed stepped-frequency continuous wave (SFCW) GPR system, which is a frequency domain sampling system with improved performance in dynamic range, sensitivity, and anti-interference ability. Because the regularly used time-varying gain function for compensating the attenuation of impulse GPR data does not make full use of the advancement of the SFCW modulation, an alternative procedure is proposed herein. The new approach is based the SFCW mechanism, and aims at improving the visualization of deeper targets by compensating the SFCW GPR signal attenuation. We first present the attenuation mode of the SFCW GPR echo, from which an inverse attenuation function is derived to compensate the amplitude loss. For the field measurement where the theoretical inverse attenuation function is difficult to achieve, we introduced a pseudo time–frequency distribution for estimating the inverse attenuation function. A procedure for amplitude attenuation has also been developed. Testing with both synthetic and experimental data return a good reconstruction of the signal amplitude, subsequently improving the ability for visualizing and detecting deeper targets.

## 1. Introduction

Ground penetrating radar (GPR) is widely used in geophysical, environmental, and civil engineering applications as a non-destructive sensor. There are two common categories of GPR: impulse and continuous wave [1]. Most GPR systems are based on the impulse radar technique and are prevalent in the commercial market. Because the stepped-frequency continuous wave has many advantages such as wider dynamic range, greater sensitivity, and higher immunity to radio frequency interference [2,3], the design and implementation of stepped-frequency continuous wave (SFCW) GPR is becoming increasingly popular [3,4,5,6,7,8,9]. Recent development in electronic devices also remove the obstacles to implementing the SFCW GPR architecture [10].

In the measurement, SFCW GPR systems transmit a continuous wave with a frequency that is stepped in linear increments over a fixed bandwidth, from a start frequency to a stop frequency. The received signal is sampled at each discrete frequency step, and then the digitized waveform is transformed into the time domain by inverse discrete Fourier transform (IDFT) to create the synthesized pulse [11]. Several advantages of the SFCW GPR are the controlled transmission frequencies, efficient use of power, and efficient sampling of wideband signals. The nature of the system architecture allows the collection of coherent (real and imaginary) data, which allows complex processing and the implementation of Synthetic Aperture Radar (SAR) algorithms [5], and other approaches relying on the accuracy of phase such as velocity estimation [12], migration [13], etc.

With these advantages, SFCW GPR systems are seeing more and more applications [14,15,16]. The information contained in the signal (e.g., phase, travel time, waveform, and amplitude) enable several approaches to non-invasively sense and/or monitor underground infrastructure [3,4], concrete [6,17,18], bridge decks [19], and roads [20,21]. Among all the applications, the sensing of deep underground targets and soil structure in a lossy environment is in high demand, especially for early geological-disaster warning [22,23,24,25,26]. In a lossy medium, an electromagnetic wave with higher frequency decays more rapidly than one with lower frequency [11], meaning that the greater the required penetration depth, the lower the frequency that should be considered. However, a lower frequency implies a sacrifice of resolution, resulting in missing or blurred targets with small geometry and the subtle soil structure, whereas higher frequency ensures a high resolution but performs poorly in penetrating depth. Considering that electromagnetic waves decay exponentially with the increase of depth in a lossy medium [27], the amplitude of the returns from deep targets will decrease to too low a level for reliable detection and identification of targets. Therefore, it is necessary to compensate for the signal attenuation so that we can improve the visualization of deeper targets, especially when a high frequency is used for maintaining the resolution.

Attenuation compensation for the classic impulse GPR system is usually performed with a time-varying gain function, including exponential gain, auto gain control (AGC), spreading and exponential correction (SEC), etc. [28]. Although these methods provide a promising effect for impulse GPR, they do not fit the signal mode of SFCW GPR [1]. Time-varying gain is also not able to effectively compensate the frequency-dependent attenuation. Until now, obtaining GPR data has relied more on experience, and no universal criterion is available. 

In contrast to the classic impulse GPR system, SFCW GPR samples the data for each frequency separately with a coherent mechanism. Therefore, the compensation of the signal loss can be achieved in ways that are more flexible. At the hardware level, the low noise amplifier (LNA) and the matched filter (MF) in the receiver can be designed and optimized individually for each sampling frequency band, so that the amplification chain can match with the received signal over the whole frequencies and efficiently compensate the signal loss [10,29]. In view of data processing, considering that the SFCW GPR transmits a single-frequency continuous sinusoidal wave at each sampling moment, it is possible to precisely model the propagation of the electromagnetic wave in a lossy medium with narrowband signal principles, which enables the determination of the factors that influence the amplitude attenuation. 

In addition to improving the performance of hardware, in this work, we put effort into advancing the SFCW GPR system with a novel signal processing approach. Hereby, we introduce an attenuation compensation approach for the SFCW GPR. We first present the signal model of SFCW GPR, based on which the attenuation pattern is modeled. Then, an inverse attenuation function for compensating the signal loss is theoretically derived. Since the theoretical method is difficult to achieve in practical scenarios, we further introduced an approach to estimate the inverse attenuation function from the measured data. The novel approach is based on a pseudo time–frequency spectrum that is also defined in this work. The proposed attenuation compensation approach was verified with both synthetic and experimental data, showing a satisfactory recovery of the deep target information.

## 2. SFCW GPR Modulation

**Figure 1:**
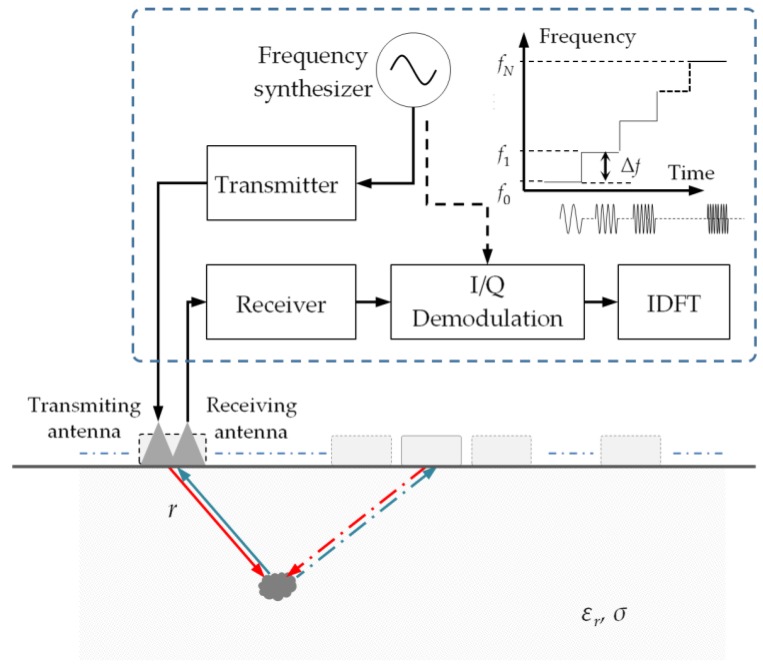
Subsurface sensing with stepped-frequency continuous wave ground penetrating radar (SFCW GPR). A diagram of the SFCW GPR is inside the dashed rectangle frame. IDFT: inverse discrete Fourier transform.

The SFCW GPR determines the distance of the target by sequentially measuring the coherent electromagnetic wave returns from the target for a series of stepped frequencies, and uses the inverse discrete Fourier transform (IDFT) to transform spatial frequency domain data into the time domain to form a synthesized pulse. Shown in Figure 1, the sensing zone is a subsurface half-space. A series of continuous sinusoidal waves that start from *f*_0_ and rise up with frequency step Δ*f* are generated sequentially by a frequency synthesizer and coupled into the medium through a wide-band antenna. The signal reflected by the target objects are subsequently received by the receiver, which is processed using quadrature demodulation [11], and finally a sequence of complex numbers (known as a trace, or A-Scan) containing the in-phase and quadrature channels is obtained for a single measurement.

By repeating this operation along the scan line, the so-called B-Scan radargram is obtained. Note that at each measurement point, the offset between the transmitting- and receiving-antenna remains unchanged, which is known as common-offset measurement. In the following, the signal model is based on the far-field approximation considering that the interested targets are far away from the antenna in deep soil. Correspondingly, the separation between the transmitting and receiving antenna can be ignored [30], and the propagation of the electromagnetic wave is modeled with a plane wave [31]. 

Assume the relative permittivity and electric conductivity of the subsurface medium are *ε_r_* and *σ*, respectively. Consider a target with radar cross section (RCS) *ς* at a distance *r* away from the antenna’s radiation center. It is illuminated by a horizontally polarized electric field $E_{x}^{T}$. The received complex electric field for a single target at the frequency *f* = *f*_0_ *+ n*·Δ*f* can be written as [11]:

(1) $$E_{x}^{R}\left(\omega ,r\right)=AE_{x}^{T}\frac{\varsigma }{r^{2}}\exp\left\{-2\alpha \left(\omega \right)r\right\}\exp\left\{-j2\beta \left(\omega \right)r\right\},$$

where *ω* = 2*πf* refers to the angular frequency and *A* is the system gain that takes the antennae and the amplification chain into account. The first exponential term in (1) characterizes the signal attenuation, whereas the second exponential term determines the phase delay. Both terms depend on the medium properties, wave frequency, and target distance. In (1), the attenuation factor *α*(*ω*) and phase factor *β*(*ω*) are defined with [11,27]:

(2) $$\{\begin{array}{c} \alpha \left(\omega \right)=\omega \sqrt{\frac{\mu \varepsilon_{a}}{2}[\sqrt{1+{\left(\frac{\sigma }{\omega \varepsilon_{a}}\right)}^{2}}-1]}\\ \beta \left(\omega \right)=\omega \sqrt{\frac{\mu \varepsilon_{a}}{2}[\sqrt{1+{\left(\frac{\sigma }{\omega \varepsilon_{a}}\right)}^{2}}+1]} \end{array},$$

where *ε_a_* = *ε_r_ε*_0_ is the dielectric permittivity and *σ* and *μ* give the electric conductivity and magnetic permeability of the medium, respectively. Consider that GPR works in a non-magnetic material. Therefore, *μ* is a constant and is equal to the permeability of vacuum. Note that *ε_a_* and *σ* can be frequency variant. Hereby, we model the medium’s dielectric properties with the frequency invariant assumption [27]. 

Given that the wavelength is small compared to the size of the target space, the sensing zone can be modeled as a set Ω with *N* dominant scattering centers [32]. Merging the reflection from all the scattering centers, we get the output of SFCW GPR as follows:

(3) $$E_{x}^{R\left(\Omega \right)}\left(\omega \right)=\sum_{n=1}^{N}AE_{x}^{T}\frac{\varsigma_{n}}{r_{n}^{2}}\exp\left\{-2\alpha \left(\omega \right)r_{n}\right\}\exp\left\{-j2\beta \left(\omega \right)r_{n}\right\}.$$

It can be seen that a measurement at frequency *ω* equals to a complex convolution of the incident electromagnetic wave with the Green’s function of the sensing area. After sweeping the frequency band, an equivalent frequency spectrum is obtained. Therefore an IDFT is performed to synthesize the time domain pulse [11]:

(4) $$E_{x}^{R\left(\Omega \right)}\left(\omega \right)\overset{\mathrm{IDFT}}{\underset{\mathrm{DFT}}{⇌}}s\left(t\right).$$

Generally, the phase components record the travel history of the wave and relate to the targets’ spatial distribution. Most data processing approaches are based on the phase information, such as FK migration [13], wave velocity estimation [12], etc. However, since the amplitude decays significantly with the increase of the frequency and distance, detecting deep targets and recovering the scattering intensity quantitatively are very difficult. This means that the attenuation of amplitude needs to be calibrated. 

## 3. Attenuation Compensation

By substituting ${AE}_{x}^{t}$_0_ with *A*_0_, and the attenuation term in (1) with

(5) $$G\left(\omega ,r\right)=\frac{\exp\left\{-2\alpha \left(\omega \right)r\right\}}{r^{2}},$$

Equation (1) is simplified to

(6) $$E_{x}^{R}\left(\omega ,r\right)=A_{0}\cdot G\left(\omega ,r\right)\cdot \exp\left\{-j2\beta \left(\omega \right)r\right\}\cdot \varsigma .$$

This shows that the output of the SFCW GPR contains three parts: the first is the system gain *A*_0_, which is a constant for the given antenna configuration and GPR setups; the second part is the attenuation function *G*(*ω*,*r*), which depends on the medium properties, wave frequency, and distance, and affects the amplitude of the received data; the last part presents the phase delay, which is also determined by the properties of the medium and the frequency of the electromagnetic wave. 

The exponential attenuation pattern in (5) implies that the amplitude of the collected signal would decrease significantly with the increase of the depth. For the impulse GPR system, a time-variant gain function can normally be applied to amplify the deeper data, such that the weak signal can be enhanced and the deep targets can be detected.

### 3.1. Signal Attenuation Mode of SFCW GPR

Expanding *α*(*ω*) with Maclaurin series, we get

(7) $$\alpha (\omega )\approx \frac{\sigma }{2}\sqrt{\frac{\mu }{\varepsilon_{a}}}\left(1-\frac{\sigma^{2}}{8\omega^{2}\varepsilon_{a}^{2}}\right).$$

Applying (7) to (6), the attenuation function is approximated to

(8) $$G(\omega ,r)\approx \frac{1}{r^{2}}\cdot \exp\left\{-\sigma \sqrt{\frac{\mu }{\varepsilon_{a}}}\cdot r\right\}\cdot \exp\left\{\frac{\sigma^{3}}{8}\sqrt{\frac{\mu }{\varepsilon_{a}^{3}}}\cdot \frac{r}{\omega^{2}}\right\}.$$

Equation (8) presents, in theory, that the attenuation mode of the SFCW GPR signal is a function of frequency and distance in the lossy medium. The higher the radar frequency is, the more severe attenuation would be, so as the distance.

### 3.2. Attenuation Compensation—Theory

Even though the higher frequency band ensures a better resolution, it suggests more severe amplitude attenuations. As a result, the amplitude of echoes from the deeper targets fall off too much and therefore reduce the visibility of the deeper targets. To solve the dilemma, the attenuation compensation approach should take into account both the frequency- and distance-dependent signal attenuation. An inverse attenuation function is firstly estimated based on the attenuation mode, and then the received data is compensated as follows:

(9) $$S\left(\omega \right)=\Psi \left(\omega \right)\cdot E_{x}^{R\left(\Omega \right)}\left(\omega \right),$$

where Ψ(*ω*) is the inverse attenuation function. In theory, for the single target scenario, a theoretical inverse attenuation function can be built from (8) with

(10) $$\Psi \left(\omega \right)=G^{-1}\left(\omega ,r\right)\approx r^{2}\cdot \exp\left\{\sigma \sqrt{\frac{\mu }{\varepsilon_{a}}}\cdot r\right\}\cdot \exp\left\{-\frac{\sigma^{3}}{8}\sqrt{\frac{\mu }{\varepsilon_{a}^{3}}}\cdot \frac{r}{\omega^{2}}\right\}.$$

However, analytic expressions like (10) are not applicable because the properties of the medium including the permittivity, conductivity, and the distance of the target are all unknown. Considering that SFCW GPR works with the frequency-division mode, hereby we proposed a pseudo time–frequency spectrum that is used to estimate the inverse attenuation function. 

The pseudo time–frequency transform (PTFT) is defined as follows:

(11) $$\Gamma_{x}\left(t,\omega \right)=\frac{1}{2\pi }\int x\left(\upsilon \right)g_{t,w}^{*}\left(\upsilon -t\right)\exp\left\{j\omega t\right\}d\upsilon ,$$

where *x*(*υ*) is the input, and the *g_t,w_*(*υ*) is a symmetric window function that meets $\left\|g_{t,w}\left(\upsilon \right)\right\|=1$. The pseudo time–frequency transform is similar to the classic time–frequency transform [33], but uses a complex-exponential kernel with opposite sign, and includes the factor 1/2π. Note that when *g_t,w_*(*υ*) = 1, Equation (11) collapses to the inverse Fourier transform. In this paper, a normalized Hamming window is adopted as *g_t,w_*(*υ*). 

Since the SFCW GPR works as a frequency sampling system, when performing the pseudo time–frequency transform on the SFCW GPR data, the input is actually a frequency spectrum and the obtained pseudo time–frequency spectrum is a joint frequency–time distribution. For maintaining the context consistency, in the following we still use the wording “time–frequency”.

**Figure 2:**
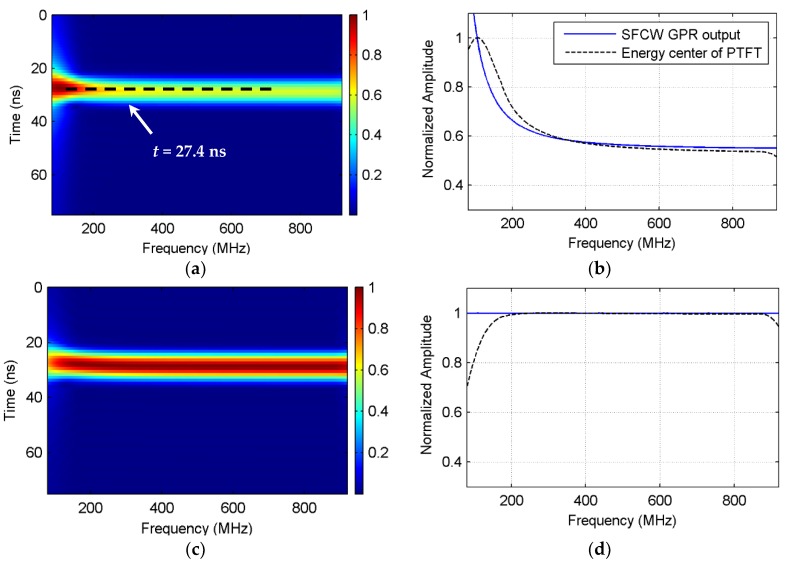
Pseudo time–frequency distribution of the synthetic SFCW GPR data from a scatter locates at 2 m away from the antenna center in the medium with a relative permittivity of 5 and electric conductivity of 25 mS/m. (**a**) is the normalized pseudo time–frequency spectrum, where a dashed line indicates the time lag corresponding to the target; (**b**) shows the spectrum profile at 27.4 ns; (**c**,**d**) present the compensated spectrum corresponding to (**a**,**b**), respectively. PTFT: pseudo time–frequency transform.

As an example, Figure 2a illustrates a pseudo time–frequency spectrum for the SFCW GPR data simulated with a theoretical scattering point that is 2 m away from the surface with unity RCS. The subsurface was set to a high lossy medium with a relative permittivity of 5 and electric conductivity of 25 mS/m. In Figure 2a, an apparent line spectrum indicated by black dash line is noticed centered at 27.4 ns (corresponds to a distance of 2 m). The energy center of the line spectrum presents an exponential attenuation pattern as the frequency increases, as shown in Figure 2b (black dash line). This pattern is similar to the output of SFCW GPR (blue line in Figure 2b), which implies that the pseudo time–frequency transform actually decomposes the overlaid echo back to each time window. This is consistent with the typical time–frequency theory. 

**Figure 3:**
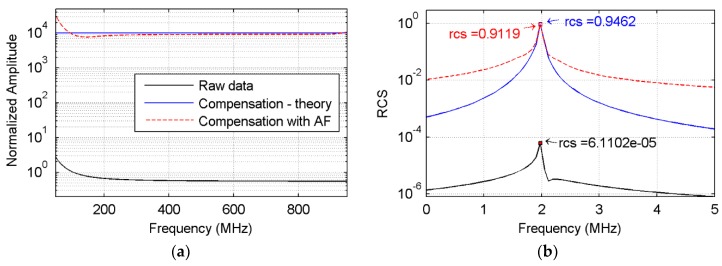
Attenuation compensation of point scatter. (**a**) Compares the collected data (black line), the data compensated with the theoretical inverse attenuation function (blue line), and the estimated inverse attenuation function (red-dashed line); (**b**) displays the corresponding time domain pulse. Note that the *y*-axis in both (**a**,**b**) are in algorithm mode for a better visual effect. RCS: radar cross section.

The assumption is that if the attenuation terms in (6) can be removed, the only factor that determines the amplitude is the target’s RCS. Performing the pseudo time–frequency transform on this ideal echo, we got a pseudo time–frequency spectrum that shows a horizontal distribution pattern (Figure 2c), and the center profile became a flat window (Figure 2d). Inspired by this assumption, hereby we estimate the inverse attenuation function by reversing the envelope of the spectrum obtained at the time corresponding to the center of the line spectrum on the pseudo time–frequency representation. Note that near the low- and high-frequency bands of Figure 3, the tapering phenomenon is observed, caused by the window effect when performing the transform.

With the pseudo time–frequency transform, the attenuation compensation works as follows: the measured SFCW GPR data are transformed to the pseudo time–frequency representation with PTFT, then the line spectrum at the time lag corresponding to the target’s distance is determined by finding the maximum energy center, whose envelope is taken out as the approximated attenuation pattern. Next, an inverse attenuation function is estimated by reversing the envelope, and multiplies the measured data. In this way, the attenuation compensation is finished.

The effect of the attenuation compensation was tested with the simulation, as in Figure 2a,b. The output of SFCW GPR (black line in Figure 3a) was compensated with the theoretical and estimated inverse attenuation functions, separately, which are shown with blue and red dashed lines in Figure 3a. Accordingly, the time-domain impulse responses were obtained using the IDFT and are presented in Figure 3b. All the responses showed a peak value around the real distance of 2 m. When taking the pulse intensity as the estimation for the RCS, it can be seen that the RCS without compensating the attenuation was only 6.11 × 10^−5^, much lower than the real value. Performing attenuation compensation using the estimated inverse attenuation function, the recovered RCS value was 0.9119, which is close to the theoretical result of 0.9462. The result indicates a good recovery of the target RCS and distance.

Nevertheless, due to the limited bandwidth and the approximations adopted for estimating the attenuation function, there were still misfits between the theoretical data and the data compensated with the estimated inverse attenuation function. Misfit values also existed at the beginning and end of the spectrum, and the obtained RCS was also lower than the real value. However, the good recovery of both distance and RCS shows that the error was at an acceptable level and can be improved using a wider bandwidth and a higher frequency. 

### 3.3. Attenuation Compensation for the Real Scenario

A practical sensing zone can be modeled with a set of dominant scattering centers. Therefore, the collected signal with SFCW GPR is a vectorial accumulation of the reflections from all the scattering points. Because the attenuation depends directly on the distance, it is not applicable to estimate a common inverse attenuation function that can fit all the attenuation modes corresponding to the targets with various distances. 

Considering that the reflections from different targets possess a separated time delay, and that the objects in the real scenario are not continuously distributed, this would yield separated spectrum lines in the pseudo time–frequency spectrum [33]. Consequently, herein we explored the possibility of compensating the echo from different targets with a separated inverse attenuation function based on the CLEAN technique [34]. 

**Figure 4:**
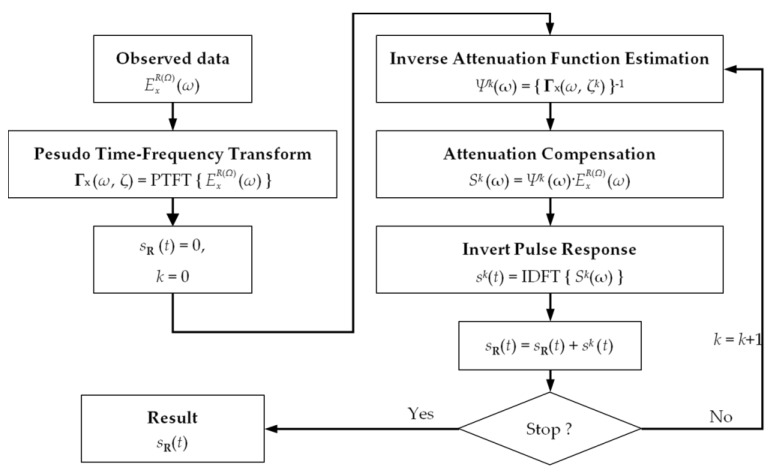
Scheme of using the CLEAN tech to compensate the attenuation function.

Figure 4 gives the scheme to compensate the attenuation for the multi-target scenario. In the first step, the measured frequency domain sequence is transformed to pseudo time–frequency spectrum with (11). The compensated sequence *s_R_*(*t*) and time instant index *k* are reset. The attenuation compensation is performed with an iterative operation. At the *k*th iteration, a line spectrum Γ*_x_*(*ω*, *ζ^k^*) is extracted as the frequency-dependent attenuation corresponding to the travel time of *ζ^k^*, by inverting which a local inverse attenuation function *Ψ^k^*(*ω*) is estimated. The operation is based on the fact that a pulse with travel time *ζ^k^* would introduce a line spectrum in the pseudo time–frequency spectrum around also the time instant *ζ^k^*, which can represent the frequency-dependent attenuation.

Next, the SFCW GPR data are compensated with (9), and then the compensated data are transformed to the time domain with the IDFT, forming a pulse *s^k^*(*t*) and are accumulated to the output *s*_R_(*t*). The iteration is continued until all the time instants are traversed. Then, *s*_R_(*t*) gives the final result.

**Figure 5:**
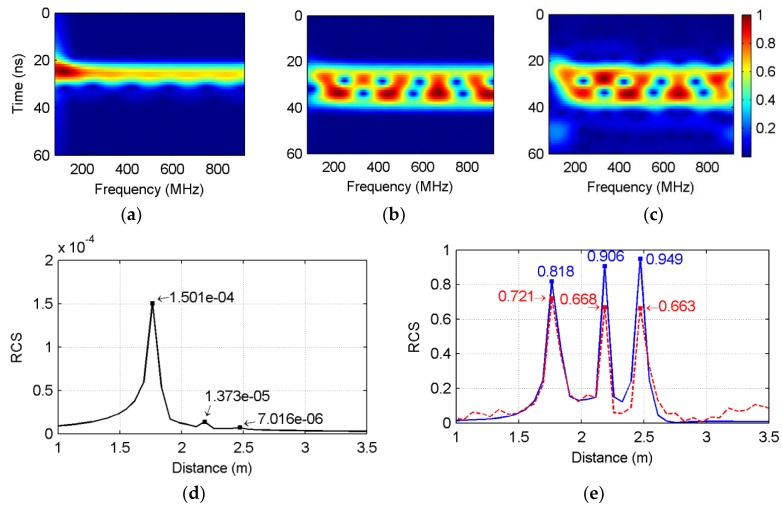
Attenuation compensation for the multi-target scenario. Three targets located at 1.8 m, 2.2 m, and 2.5 m were simulated and tested for the proposed attenuation compensation approach, where (**a**–**c**) show the pseudo time–frequency spectra for the raw data, the data with ideal no-attenuation assumption, and the data compensated with the estimated inverse attenuation function; (**d**) is the raw pulse response before compensating the amplitude attenuation; whereas (**e**) illustrates the theoretical pulse response with the blue line, and the one calibrated with the estimated inverse attenuation function. Note that (**d**,**e**) have a different range for the *y*-axis, and (**a**,**c**) use normalized amplitude for better comparison.

The proposed approach was tested with a scenario containing three targets located at 1.8 m, 2.2 m, and 2.5 m away from the antenna, and all were with unity RCS, while keeping the medium environment the same as in Figure 3 (*ε_r_* = 5, *σ* = 25 mS/m). The echo was transformed to the time domain with IDFT (Figure 5d), and the RCS for the three targets before compensating the attenuation was 1.501 × 10^−4^, 1.373 × 10^−5^, and 7.016 × 10^−6^, respectively. The low RCS value was caused by the exponential attenuation mode. Besides, the echo pulse corresponding to the second and third targets was concealed in the background, and only the first target was identified. A similar observation was also obtained in the pseudo time–frequency spectrum shown in Figure 5a, where the line spectra relating to the second and third targets are covered by the first target.

Again, we calculated the output of SFCW GPR for the three targets without including the attenuation. The theoretical echo in the time domain presents as three pulses centered at 1.8 m, 2.2 m, and 2.5 m, with pulse intensity of 0.818, 0.906, and 0.949, respectively (see Figure 5e, blue line). Meanwhile, the pseudo time–frequency spectrum under the ideal condition shows a wide line spectrum (Figure 5b). Compared to Figure 5a, the ideal pseudo time–frequency spectrum with no attenuation considered displays several interferential line spectrums. 

After compensating the attenuation with our proposed procedure, the pulse responses of the three targets can be clearly observed in Figure 5e (red dash line), where three pulses are well observed and comparable to the result obtained from the theoretical calculation. Correspondingly, the pseudo time–frequency spectrum (see Figure 5c) was also improved and presents similar distribution to the theoretical one shown in Figure 5b. 

Note that the process was carried out for one measurement (i.e., the A-scan). For a B-Scan that contains more than one measurement point, attenuation compensation is done trace-by-trace. For the RCS misfit between the real and the estimated values, it is expected to be due to the approximations and numerical calculation losses. Actually, for most measurements, a relative RCS distribution is sufficient for subsurface sensing, and thus the proposed approach would be able to fit most SFCW GPR applications.

## 4. Experimental Results

**Figure 6:**
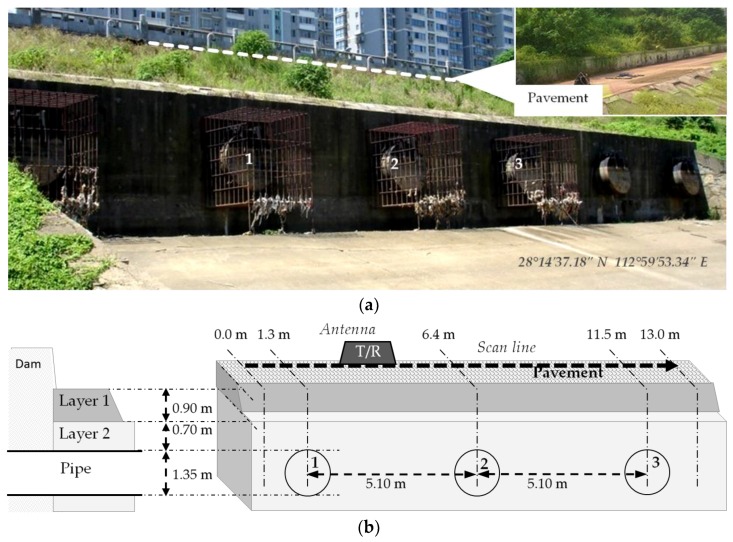
The measurement site. (**a**) A photo of the measurement site. On top of the site, there was a pavement on which we carried out the measurement. The number indicates the target pipes; (**b**) gives the schematic of the measurement. T/R indicate transmitting and receiving antennae, respectively.

We used the novel developed approach with a real SFCW GPR system [35]. The field measurement was carried out over the drainage pipes of a pump facility near the Liuyang River of Changsha, China (28°14′37.18″ N, 112°59′53.34″ E), where six rainwater releasing pipes are buried beneath the pavement along the bank of the river, as shown in Figure 6. The soil over the pipes is a two-layer structure, where the top layer with a thickness of 0.90 m is the base of the pavement, and the bottom layer with concrete shelter holds the pipes. The soil on the top of the pipe in the second layer is 0.70 m thick, which means the SFCW GPR needs to sense the target under 1.6-m-thick two-layer soil. As illustrated in Figure 6b, the measurement was done along a 13.00-m-long scan line perpendicular to the extending direction of the pipe. Three pipes denoted with numbers in Figure 6a were under the scan line. All three pipes had the same diameter of 1.35 m. 

The frequency range was 50 MHz–1 GHz stepped by 1.73 MHz, meaning a 551-frequency-point sequence for each measurement position. Unshielded bowtie antennae were used as the transmitting and receiving antennae. The horizontal distance between two measurements was 0.10 m, and a total of 130 traces were obtained. For reliability, we repeated the measurement three times. 

**Figure 7:**
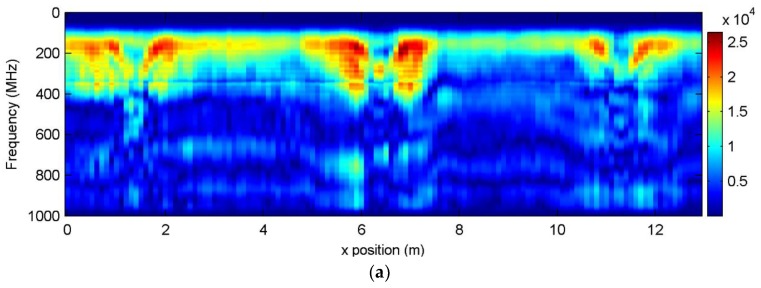

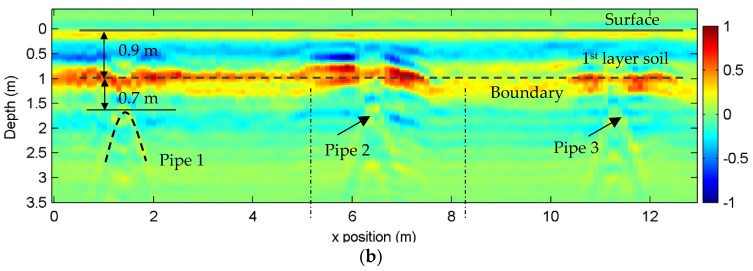
Field measured data with SFCW GPR. (**a**) The measured SFCW GPR data, where each column is a measurement and the 130 measures are stacked along the *x*-direction forming a B-scan profile; (**b**) Spatial domain data obtained with IDFT. The wave velocity 0.13 m/ns was used to convert the travel time to the depth. The horizontal solid line indicates the surface, whereas with the horizontal dashed line we mark the boundary between the first and second layers of soil.

The 130 traces were stacked together and formed the B-scan profile shown in Figure 7a, where each column represents a measurement. Three areas with high-energy intensity centered at 1.3 m, 6.4 m, and 11.5 m were observed, corresponding to the pipes’ locations. Performing the IDFT for each column (i.e., the pulse compression), we obtained the radargram shown in Figure 7b.

The radar profile in Figure 7b presents a two-layer structure, where the thickness of the first layer is around 0.9 m with the estimated wave velocity of 0.13 m/ns (corresponding to the relative permittivity of 5.4, a typical value for soil). With careful inspection, the hyperbolic pattern for the reflection from cylindrical objects can be detected in Figure 7b. Note that here the data is not enhanced with a gain function. Due to the large depth and high dielectric loss in the multi-layer soil, the observed reflection was too weak for reliable detection and recognition. The boundary reflection also covered the pipes, making the data interpretation more difficult. In the next step, we used the attenuation compensation approach to improve the data. 

The signal attenuation was compensated column-by-column with the procedure given in Figure 4. For each trace, the inverse attenuation function was estimated and compensated with the depth interval Δ*d* = 0.032 m (corresponding Δ*t* is 0.5 ns). Since the depth of the target of interest was between 1.5 m and 3.0 m, we set the attenuation compensation stop at 3.5 m (54 ns) to save on computing time. All 130 traces were processed in 11.22 s (with Intel I7 CPU and 8 GB memory). 

**Figure 8:**
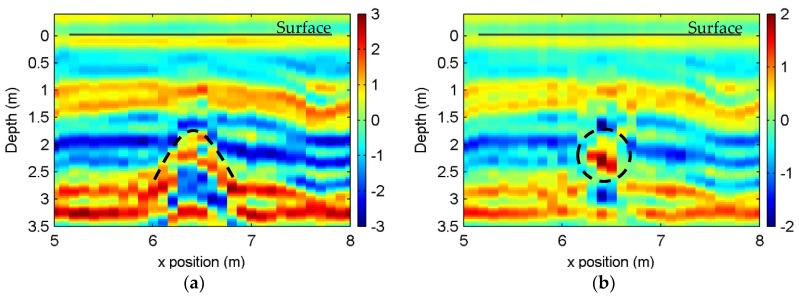
(**a**) is the radar profile between 5 m and 8 m after compensating the attenuation; (**b**) shows the imaging result for (**a**), where the dashed circle marks the real position of pipe 2. In both (**a**,**b**), the horizontal solid line indicates the surface.

Figure 8a presents the data after the attenuation compensation between 5 m and 8 m. With attenuation compensation, the data were improved in two aspects: the hyperbola pattern for the target was clearer, and the relative amplitude fit the real environment. Furthermore, we obtained the image (Figure 8b) of the part shown in Figure 8a using the TAM-BP imaging algorithm [36], which aims at imaging layered soil structure. The target distribution and layered texture in Figure 8b show a good recovery of the sensing zone. 

## 5. Discussion and Conclusions

This work developed a novel approach with an attenuation compensation solution to compensate the signal loss of SFCW GPR data, subsequently calibrating the amplitude of the signal corresponding to deep targets and making deep targets more visible. The work began with a theoretical analysis of the signal attenuation. With a single point reflection model, we first derived the attenuation mode in a lossy medium, and then proposed a method of compensating the attenuation by introducing the inverse attenuation function. Since the inverse attenuation is difficult to obtain in reality, we further introduced the pseudo time–frequency transform. Finally, we accomplished the attenuation compensation approach with an iterative operation for the real scenario that is modeled with a set of dominant scattering point-centers.

The results showed that the recovered RCS for the synthetic case fit with preset model. Note that even though the obtained RCSs in the multi-target scenario were lower than the real value, they presented similar magnitudes. This is useful for real observation, where the contrast of different targets and/or layers is of more concern. For the experiment, the compensated data showed a promising result in revealing the subsurface soil structure and the pipes’ reflections, which were previously very weak due to the large depth and covered by the boundary reflection. A third layer boundary beneath the pipe (Figure 8) was also observed. Based on its location, we suggest that this extra boundary indicated the bottom of the facility. Note that because the used SFCW GPR is a test system with limited radiation power, and the moisture soil near the river introduces high loss, the effective observation depth is insufficient for more complicated applications, such as early disaster warning. Future work consists of including detailed antenna effects and combination with more complex medium environments.

Compared to the classic gain approaches, the proposed attenuation compensation method is derived and developed based on the properties of stepped-frequency and continuous wave mechanisms. In cooperation with the time–frequency distribution theory and phase spectrum, further advancement is expected in the next work.
